# Clinical and Radiological Characteristics of Symptomatic Emphysema Patients with PRISm and Pre-COPD Phenotypes: Possible Effects of Smoking Status

**DOI:** 10.3390/biomedicines14061245

**Published:** 2026-05-30

**Authors:** Maşide Ari, Emrah Ari, Eray Çinar, Hakan Ertürk, Deniz Çelik, Murat Yildiz, Tarkan Özdemir, Mehmet Kayadelen, Derya Tüten Özdemir, Tunahan Dolmuş, Hasan İbiş, Esma Dolmuş, Ömer Faruk Tüten

**Affiliations:** 1 Department of Pulmonology, Ankara Atatürk Sanatorium Training and Research Hospital, 06290 Ankara, Turkey; drmuratyildiz85@gmail.com (M.Y.); kydln@hotmail.com (M.K.); hsanibis@gmail.com (H.İ.); 2 Department of Emergency Medicine, Mamak Public Hospital, 06620 Ankara, Turkey; dremrahari25@gmail.com; 3 Department of Thoracic Surgery, Bilkent City Hospital, 06800 Ankara, Turkey; ecinar36@gmail.com; 4 Department of Radiology, Ankara Atatürk Sanatorium Training and Research Hospital, 06290 Ankara, Turkey; ert.hakan@gmail.com; 5 Department of Pulmonology, Faculty of Medicine, Alanya Alaaddin Keykubat University, 07450 Antalya, Turkey; drdenizcelik@hotmail.com; 6 Department of Pulmonology, Konya Farabi Hospital, 42090 Konya, Turkey; tarkanozdemir78@gmail.com; 7 Department of Thoracic Surgery, Ahi Evren Thoracic and Cardiovascular Surgery Training and Research Hospital, 61040 Trabzon, Turkey; deryatuten@gmail.com; 8 Department of Pulmonology, Gebze Fatih Public Hospital, 41400 Kocaeli, Turkey; 9 Department of Pulmonology, Darıca Farabi Training and Research Hospital, 41700 Kocaeli, Turkey; esma.zenbilli@gmail.com; 10 Department of Pulmonology, Faculty of Medicine, Ankara University, Health Practice and Research Hospitals, 06230 Ankara, Turkey; omertuten@gmail.com

**Keywords:** emphysema, HRCT, pre-COPD, PRISm, smoking status, non-tobacco exposure, quantitative CT

## Abstract

**Background:** Pre-Chronic Obstructive Pulmonary Disease (pre-COPD) and Preserved Ratio Impaired Spirometry (PRISm) phenotypes represent important components of the early obstructive lung disease spectrum, characterized by respiratory symptoms and structural lung abnormalities prior to the development of overt airflow limitation. Emphysema is considered one of the major structural phenotypes underlying airway disease and the COPD spectrum. Although cigarette smoking is the best recognized risk factor for these conditions, non-tobacco exposures may also contribute to early structural lung changes. In this study, we evaluated the radiological features, pulmonary function parameters, and dyspnea severity of CT-detected emphysema in symptomatic patients classified as having pre-COPD or PRISm, with particular attention paid to the potential influence of smoking status on disease characteristics. **Methods:** In this retrospective, single-center study, symptomatic patients aged 20–50 years classified as having pre-COPD or PRISm and in whom emphysema was detected on high-resolution computed tomography (HRCT) were evaluated. Only symptomatic patients who underwent HRCT for clinical indications and in whom emphysema was identified were included. Demographic characteristics, emphysema type and quantitative emphysema severity, pulmonary function parameters, and Modified Medical Research Council (mMRC) dyspnea scores were analyzed. The PRISm and pre-COPD groups were compared in terms of clinical and symptomatic characteristics. In addition, smoking-related clinical and radiological characteristics were also evaluated. **Results:** A total of 232 patients were included in the study. The median age was 43 years (38–48), and 84.1% of the participants were male. Among the study population, 68.5% were classified in the pre-COPD group and 31.5% in the PRISm group. The most frequently identified emphysema patterns were paraseptal (44.4%) and centrilobular (40.5%). The median total lung emphysema area was 18% (13–22). A weak negative correlation was observed between the degree of emphysema and FEV_1_ (r = −0.185; *p* = 0.005), whereas a weak positive correlation was found between emphysema extent and the mMRC dyspnea score (r = 0.214; *p* = 0.001). Dyspnea severity was significantly higher in the PRISm group compared with the pre-COPD group (*p* < 0.001). In the smoking-status subgroup analysis, ever-smokers demonstrated significantly greater dyspnea severity and lower FEV_1_ values, whereas never-smokers had a significantly higher proportion of emphysema extent > 18% (all *p* < 0.05). **Conclusions:** Radiologically detected emphysema in symptomatic patients without airflow limitation was associated with statistically significant but weak alterations in pulmonary function and dyspnea burden. Dyspnea severity was significantly higher in the PRISm phenotype. In a smoking-status subgroup analysis, ever-smokers had significantly greater dyspnea severity, whereas never-smokers showed a significantly higher proportion of extensive emphysema (>18%), despite similar functional impairment across groups. These findings underscore the importance of non-tobacco exposures in the development of emphysema within pre-obstructive spirometric phenotypes. Multicenter prospective studies incorporating healthy controls and systematic exposure documentation are needed to confirm these observations.

## 1. Introduction

Chronic obstructive pulmonary disease (COPD) is a widespread public health problem and a major cause of morbidity and mortality worldwide [1]. The disease is characterized by persistent structural alterations in the airways and alveolar structures, with emphysema representing one of its most prominent components [2]. However, recent studies have demonstrated that emphysema can also be detected radiologically in individuals without airflow limitation, with an estimated prevalence of approximately 13% in the general population [3].

Due to the heterogeneous nature of COPD, intermediate phenotypes such as Preserved Ratio Impaired Spirometry (PRISm) and pre-COPD have been increasingly recognized in recent years [4,5,6]. Although individuals with these phenotypes do not exhibit overt airflow limitations, they may present with symptoms such as dyspnea and exercise intolerance, and imaging studies—including computed tomography (CT)—may reveal emphysema or airway abnormalities [7]. These findings suggest that structural alterations in the lungs may have clinically relevant consequences even before the development of classical airflow obstruction.

The updated Global Initiative for Chronic Obstructive Lung Disease (GOLD) report emphasizes the importance of identifying individuals at risk for COPD before the development of overt airflow limitation. Within this framework, pre-COPD and PRISm are defined as clinical phenotypes in which respiratory symptoms and structural lung abnormalities may be present despite the absence of airflow obstruction [5,6]. In these phenotypes, CT imaging may reveal emphysema or airway abnormalities, and some individuals have been reported to carry an increased risk of subsequently developing COPD.

Lung function peaks between approximately 20 and 25 years of age and begins declining physiologically after 45–50 years [8]. The age range of 20–50 years therefore represents a transitional period of particular vulnerability, in which early structural changes may exert clinically meaningful effects even in the absence of overt airflow obstruction. Studies simultaneously characterizing quantitative emphysema burden, spirometric phenotype, and symptom profile in this age group remain scarce. A better understanding of these relationships may advance the early identification of individuals at risk and inform targeted preventive strategies.

Although cigarette smoking remains the most widely recognized risk factor for emphysema and COPD, a growing body of evidence indicates that non-tobacco exposures play a substantial and often underappreciated role in the development of structural lung disease, particularly in younger individuals. Indoor biomass combustion, occupational exposure to dust and fumes, outdoor air pollution, and a history of recurrent respiratory infections during childhood have all been associated with accelerated lung function decline and early emphysema formation [3,8]. In low- and middle-income country settings, including certain regions of Turkey, solid-fuel burning for heating and cooking remains prevalent and may constitute an underrecognized driver of early-stage emphysematous change. Emphysema arising through non-tobacco pathways may present with distinct radiological patterns and clinical profiles compared with smoking-related disease; yet its characteristics within the GOLD 2024 pre-COPD and PRISm phenotypic frameworks remain poorly described. Understanding how smoking status shapes the radiological and clinical phenotype of emphysema in pre-obstructive spirometric categories therefore carries both scientific and public health relevance.

In this context, this study evaluated the radiological features, pulmonary function parameters, and dyspnea severity of CT-detected emphysema in symptomatic patients classified as having pre-COPD or PRISm, with particular emphasis on the differential contribution of smoking status to emphysema characteristics and clinical outcomes.

## 2. Materials and Methods

Patients aged 20–50 years who presented to the chest diseases outpatient clinic between 1 April 2022 and 30 June 2024 with complaints of chronic cough, sputum production, or dyspnea were included in the study. According to pulmonary function test (PFT) results, patients with a normal forced expiratory volume in one second/forced vital capacity (FEV_1_/FVC) ratio who were classified as having either pre-COPD or PRISm based on FEV_1_ values were identified. High resolution computed tomography (HRCT) was performed in these patients according to clinical indications, and those in whom emphysema was detected were included in the study group. Clinical data and respiratory disease classifications were independently evaluated by two pulmonologists.

### 2.1. Definition of Pre-COPD and PRISm Phenotypes

Pulmonary function tests were performed in accordance with the standards of the American Thoracic Society/European Respiratory Society (ATS/ERS) [9]. Airflow limitation was defined as an FEV_1_/FVC ratio below 70%, and patients meeting this criterion were excluded from the study. Among patients with an FEV_1_/FVC ratio of 70% or higher, phenotypic classification was performed based on FEV_1_ values. Patients with an FEV_1_ value of 80% or greater were classified as having the pre-COPD phenotype, whereas those with an FEV_1_ value below 80% were classified as having the PRISm phenotype. Although the lower limit of normal approach is statistically preferable in younger populations, the fixed FEV_1_/FVC cutoff of 70% was used to remain consistent with the GOLD 2024 classification framework and to facilitate comparison with existing literature.

### 2.2. Exclusion of Other Causes of Dyspnea

In patients presenting with dyspnea, a comprehensive exclusion process was applied to determine whether the symptoms could be attributed to another major underlying pathology. Accordingly, conditions that could potentially cause dyspnea—including asthma, interstitial lung diseases, bronchiectasis, pleural effusion, pulmonary embolism, lung malignancies, congestive heart failure, and severe anemia—were excluded from the study.

Asthma was assessed based on patients’ clinical history, physical examination findings, and the presence of bronchodilator reversibility. The possibility of pulmonary embolism was evaluated using clinical risk assessment and was excluded by thoracic CT angiography in patients in whom further investigation was deemed necessary. Pleural effusion, bronchiectasis, interstitial lung diseases, and lung malignancies were evaluated using thoracic imaging modalities, and patients with these findings were excluded from the study. The diagnosis of heart failure was considered based on clinical evaluation and echocardiographic findings. Severe anemia was defined as a hemoglobin level of <11 g/dL in men and <10 g/dL in women on complete blood count performed at presentation, and these patients were excluded from the analysis.

These evaluations were conducted independently by two experienced pulmonologists in patients presenting to the outpatient clinic of our hospital, which serves as a referral center for respiratory diseases.

### 2.3. Radiological Image Evaluation

In this study, the presence and extent of emphysema were assessed using HRCT. HRCT was performed as part of advanced diagnostic evaluation in patients whose symptoms could not be explained by standard clinical, laboratory, and radiological assessments and who continued to experience persistent complaints. All imaging studies were obtained using a 128-slice CT scanner (Philips Healthcare, Amsterdam, The Netherlands). Scans were acquired during the inspiratory phase in the supine position and without the use of intravenous contrast material. Thin-section volumetric images were obtained and reconstructed using high-spatial-resolution algorithms. Images were evaluated in both lung and mediastinal window settings, and in addition to axial images, coronal and sagittal multiplanar reconstructions were generated.

Quantitative assessment of emphysema was performed using a densitometric analysis method. Emphysematous areas were defined as low-attenuation areas below −950 Hounsfield units on inspiratory-phase HRCT images. The extent of emphysema was expressed as a percentage (%) by calculating the ratio of these areas to the total lung volume. Quantitative measurements were performed using semi-automated lung segmentation with GE Healthcare AW Server 3.2 Ext. software (GE Healthcare, Chicago, IL, USA). The results obtained were recorded as the total lung emphysema percentage for each case.

HRCT images were independently evaluated by two experienced radiologists through the hospital’s imaging system. Discrepancies in the assessment of emphysema type and extent were resolved by consensus discussion. This assessment approach was based on the recommendations of the Fleischner Society for the quantitative evaluation of emphysema using CT [10]. In the analyses, emphysema severity was categorized into two groups according to the median value of the total lung emphysema percentage: ≤18% and >18%. This exploratory threshold was selected to divide the cohort into relatively balanced subgroups, as no validated or clinically established cutoff value for emphysema severity currently exists in this population.

### 2.4. Classification of Emphysema Types

#### 2.4.1. Centrilobular Emphysema

**Figure 1:**
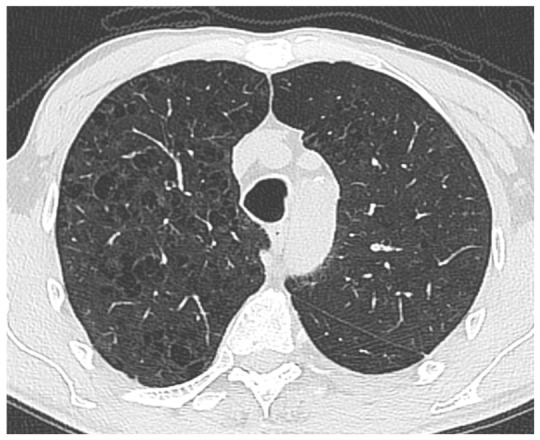
Thoracic computed tomography image demonstrating multiple small, irregularly shaped areas of low attenuation diffusely distributed in the upper lobes of both lungs. These findings are suggestive of centrilobular emphysema. The absence of a peripheral predominance and the localization of lesions adjacent to the central bronchioles are consistent with a typical centrilobular pattern.

Characterized by small, irregular areas of low attenuation that are predominantly located in the upper lobes of the lungs (Figure 1).

#### 2.4.2. Panacinar (Panlobuler) Emphysema

Defined as diffuse areas of low attenuation involving the entire lung fields.

#### 2.4.3. Paraseptal Emphysema

**Figure 2:**
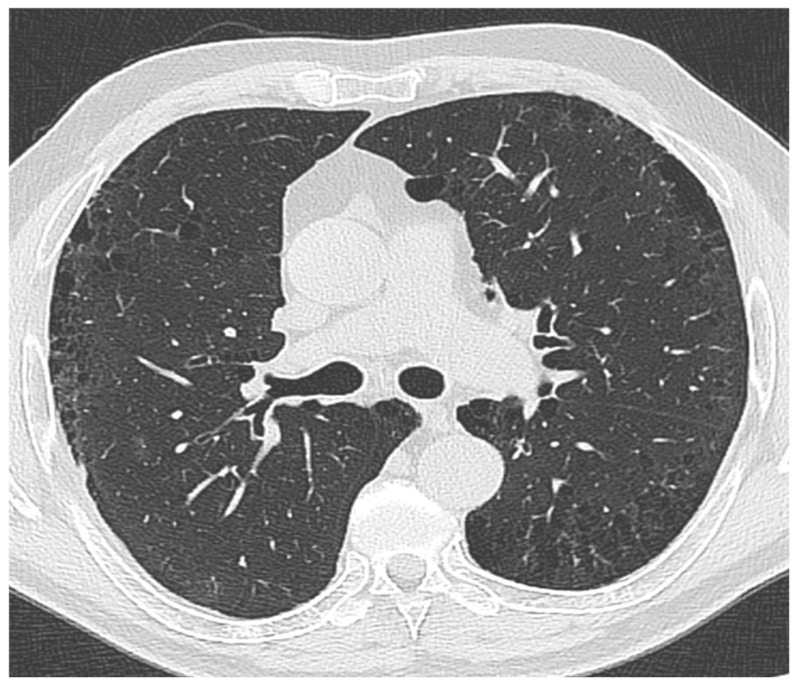
Thoracic computed tomography image of a patient included in the study demonstrating thin-walled, low-attenuation air spaces located predominantly in the subpleural regions, particularly in the upper lobes. These imaging findings are consistent with the radiological features characteristic of paraseptal emphysema.

Defined by the presence of large air-containing spaces localized in the subpleural regions (Figure 2).

### 2.5. Assessment of Body Mass Index

Body mass index (BMI) of the individuals included in the study was calculated by dividing body weight in kilograms by the square of height in meters (kg/m^2^). The obtained BMI values were categorized according to the World Health Organization (WHO) classification. Accordingly, BMI < 18.5 kg/m^2^ was defined as underweight, 18.5–24.9 kg/m^2^ as normal weight, 25.0–29.9 kg/m^2^ as overweight, and ≥30.0 kg/m^2^ as obese. This classification was used to evaluate the relationship between BMI categories, the extent of emphysema, and symptom severity.

### 2.6. Assessment of Dyspnea Severity

Dyspnea was evaluated using the Modified Medical Research Council (mMRC) scale, which is commonly used to assess the degree of breathlessness experienced by patients during daily activities [11]. The scale ranges from 0 to 4, where a score of 0 indicates dyspnea only during strenuous exercise, and a score of 4 reflects severe symptoms in which the patient is unable to leave the house or experiences breathlessness even while dressing. The mMRC scale was selected because it is a validated, widely used instrument recommended by GOLD for symptom assessment in obstructive lung phenotypes.

### 2.7. Assessment of Spirometry Quality

The acceptability and reproducibility of pulmonary function tests were evaluated according to the criteria specified in the ATS/ERS guidelines. Tests were considered unacceptable and excluded from the analysis if there was an insufficiently rapid start of the maneuver, incomplete performance of the maneuver, coughing during the test, premature termination, or inability to sustain expiration for an adequate duration.

### 2.8. Inclusion Criteria

- Patients aged between 20 and 50 years;
- Patients in whom the diagnosis of emphysema was established by a radiologist using HRCT;
- Patients presenting to the chest diseases outpatient clinic with complaints of dyspnea, cough, and/or sputum production;
- Patients classified as having pre-COPD or PRISm phenotype on pulmonary function testing;
- Patients with acceptable and reproducible pulmonary function tests;
- Patients without airflow obstruction on pulmonary function testing.

### 2.9. Exclusion Criteria

- Asymptomatic patients who did not report respiratory symptoms (dyspnea, cough, and/or sputum) at presentation;
- Individuals who underwent HRCT solely for other indications (e.g., screening, nodule follow-up, or malignancy surveillance);
- Patients for whom PFT data were unavailable;
- Patients with unacceptable PFT quality;
- Patients with airflow obstruction on pulmonary function testing;
- Patients with a history of asthma;
- Patients with radiological findings suggestive of interstitial lung disease;
- Patients with a diagnosis of lung malignancy or with radiologically suspicious pulmonary masses;
- Patients with any parenchymal lung disease;
- Patients younger than 20 years;
- Patients older than 50 years.

### 2.10. Ethical Approval

Ethical approval for this study was obtained from the Clinical Research Ethics Committee of Ankara Atatürk Sanatorium Training and Research Hospital (decision no. 159, dated 13 November 2024). The study was conducted in accordance with the ethical principles outlined in the Declaration of Helsinki.

### 2.11. Statistical Analysis

Statistical analysis of the collected data was performed using the IBM SPSS Statistics version 27.0 software package. The distribution of continuous variables was assessed using the Kolmogorov–Smirnov test. Continuous variables that did not follow a normal distribution were expressed as median (interquartile range, IQR: 25th–75th percentile). Categorical variables were presented as frequencies and percentages (%). Comparisons of clinical, demographic, and PFT variables between the two groups defined according to emphysema severity (≤18% and >18%) were performed using the Mann–Whitney U test. The chi-square test was used for comparisons of categorical variables. The relationships between the degree of emphysema and pulmonary function parameters (FEV_1_, FVC, FEV_1_/FVC, and MEF 25–75 [maximal expiratory flow at 25–75% of FVC]) as well as the mMRC dyspnea score were evaluated using Spearman correlation analysis. In addition, the PRISm and pre-COPD groups were compared with respect to clinical and symptomatic characteristics. A *p*-value < 0.05 was considered statistically significant in all analyses. Ninety-five percent confidence intervals (CIs) for Spearman correlation coefficients were calculated using bootstrapping with 1000 iterations. An exploratory subgroup analysis comparing ever-smokers and never-smokers was performed with respect to emphysema type, emphysema extent, pulmonary function parameters, and mMRC dyspnea scores. For between-group comparisons, continuous variables were compared using the Mann–Whitney U test and categorical variables using the chi-square test. Where expected cell counts were insufficient for the chi-square test, the Fisher-Freeman-Halton exact test was applied.

## 3. Results

A total of 232 patients who met the study eligibility criteria were included in the analysis. The median age of the participants was 43 years (IQR: 38–48), and the majority of the patients were male (84.1%). Of the study population, 68.5% were classified as pre-COPD and 31.5% as PRISm. The clinical and demographic characteristics of the participants are presented in Table 1. No patient in the study population had an mMRC dyspnea score of 3 or 4, consistent with the relatively young age and moderate emphysema burden of this cohort.

The emphysema types, quantitative emphysema ratios, and PFT parameters of the patients are presented in Table 2. The most frequently observed emphysema patterns were paraseptal and centrilobular types, and the median total lung emphysema percentage was found to be 18% (Table 2).

Among the 28 patients with a history of pneumothorax, evaluation of emphysema types revealed that paraseptal emphysema was the most frequently observed pattern (n = 17, 60.7%). This was followed by centrilobular emphysema (n = 10, 35.7%).

When patients were divided into two groups according to total lung emphysema extent (≤18% and >18%), significant differences were observed between the groups in terms of BMI distribution (*p* = 0.001), dyspnea severity assessed by the mMRC scale (*p* = 0.007), and FEV_1_ values (*p* = 0.023). In contrast, no significant differences were found between the groups with respect to age, sex, smoking history, presence of comorbidities, or other PFT parameters (*p* > 0.05) (Table 3).

In the correlation analysis, a statistically significant but weak negative correlation was observed between emphysema extent and FEV_1_ (r = −0.185; 95% CI: −0.305 to −0.058; *p* = 0.005), and a statistically significant but weak positive correlation was found between emphysema extent and mMRC dyspnea score (r = 0.214; 95% CI: 0.085 to 0.337; *p* = 0.001). No significant correlations were observed for FEV_1_% predicted, FVC, FEV_1_/FVC, or MEF 25–75.

When the PRISm and pre-COPD groups were compared, no significant differences were observed between the groups in terms of age, smoking history, sex distribution, BMI, presence of comorbidities, or emphysema severity (*p* > 0.05). In contrast, dyspnea severity (mMRC) was significantly higher in the PRISm group (*p* < 0.001) (Table 4).

An exploratory subgroup analysis comparing ever-smokers (n = 162, 69.8%) and never-smokers (n = 70, 30.2%) was performed. Dyspnea severity was significantly greater among ever-smokers (*p* = 0.026). In contrast, the proportion of patients with emphysema extent >18% was significantly higher in never-smokers compared with ever-smokers (55.7% vs. 38.3%, *p* = 0.014). FEV_1_ values (in liters) were significantly lower in ever-smokers (*p* = 0.002). No significant differences were observed between the groups with respect to emphysema subtype distribution (*p* = 0.051), FEV_1_% predicted, FVC, FEV_1_/FVC ratio, BMI, comorbidity prevalence, or history of pneumothorax. For the analysis of emphysema subtype distribution, the Fisher-Freeman-Halton exact test was applied owing to small expected cell counts. The detailed results of this comparison are presented in Table 5.

## 4. Discussion

This study is among the limited number of investigations evaluating the clinical and functional implications of radiologically detected emphysema in young adults without airflow limitations. Emphysema is most commonly studied in older populations and in association with obstructive airway diseases. Our results indicate that CT-detected emphysema in this population is associated with statistically significant but weak alterations in FEV_1_ and dyspnea severity, highlighting the potential clinical relevance of structural lung findings prior to overt airflow obstruction. The modest effect sizes underscore that emphysema extent alone does not fully account for the observed symptom burden. The PRISm phenotype was associated with significantly greater dyspnea compared to pre-COPD despite similar emphysema extent between groups, supporting the need for careful clinical evaluation of this patient population. Notably, the exploratory comparison between ever-smokers and never-smokers revealed distinct clinical and radiological profiles: ever-smokers had significantly greater dyspnea burden, whereas never-smokers demonstrated significantly higher emphysema extent, highlighting the differential contribution of smoking and non-tobacco exposures to disease expression in this population.

The literature indicates that emphysema is not solely a pathology confined to the respiratory system but may also be associated with metabolic and systemic disorders such as hypertension, cardiovascular diseases, and diabetes [12,13]. Similarly, systemic comorbidities have been reported to occur frequently in early phenotypes of obstructive lung disease, including PRISm and pre-COPD, and this condition may be particularly associated with an increased burden of cardiovascular and metabolic diseases [14,15]. In our study, at least one comorbidity was identified in 26.7% of the evaluated patients, with cardiovascular diseases, hypertension, and diabetes mellitus being the most commonly observed conditions. These findings support the notion that systemic comorbidities may be present not only in advanced COPD but also in early disease phenotypes.

The PRISm phenotype is defined as a clinical pattern associated with reduced pulmonary function, increased symptom burden, and adverse long-term clinical outcomes [16]. In the literature, the prevalence of this phenotype has been reported to range between 7% and 20% across different populations. Male sex, smoking, and metabolic comorbidities are among the main characteristics associated with PRISm [17]. In our study, similarly, the majority of the study population consisted of male individuals, and no significant differences were observed between the PRISm and pre-COPD groups in terms of sex distribution or comorbidity prevalence. However, the higher proportion of individuals with a smoking history of ≥20 pack-years in the PRISm group may suggest that smoking-related structural lung damage could be more pronounced in this phenotype. In addition, although the proportion of overweight and obese individuals was higher in the PRISm group in our study, this difference did not reach statistical significance. Previous studies have likewise reported that the PRISm phenotype may be associated with obesity and metabolic comorbidities [18].

One of the clinically notable features of the PRISm phenotype is the presence of a substantial symptom burden. Previous studies have demonstrated that patients with PRISm may, in some cases, experience symptom levels comparable to—or even greater than—those observed in individuals with COPD [19]. In the study by Evans et al., it was reported that 57% of patients with PRISm had an mMRC score ≥ 2, indicating a considerable dyspnea burden [20]. In our study, the proportion of patients with an mMRC score of 2 was 23.3% in the PRISm group compared with 5.7% in the pre-COPD group. Similarly, dyspnea severity was found to be significantly higher in the PRISm group. These findings suggest that the PRISm phenotype may represent not only a spirometric classification but also a clinically relevant condition characterized by a notable symptom burden.

Quantitative radiological assessment of emphysema is an important approach for elucidating the relationship between structural lung changes and clinical as well as functional findings. Several studies in the literature have demonstrated significant associations between the extent of emphysema and pulmonary function parameters. In a study conducted by Senel et al. in patients with COPD, emphysema severity was reported to be significantly associated with FEV_1_ levels [21]. Similarly, in a large COPD cohort analyzed by Kahnert et al., significant relationships were identified between CT-determined emphysema burden and spirometric parameters [22]. In our study, consistent with these findings, a weak but statistically significant negative correlation was observed between emphysema severity and FEV_1_. In addition, a significant positive association was found between emphysema severity and the mMRC dyspnea score. These findings suggest that structural lung changes in symptomatic individuals may be related not only to pulmonary function parameters but also to clinical symptom burden. Furthermore, with regard to emphysema subtypes, the predominance of paraseptal emphysema in this young patient population represents one of the notable findings of our study. In the literature, paraseptal emphysema has often been associated with spontaneous pneumothorax and is reported to occur more frequently in young men [23]. In this context, the observation that more than half of the patients with a history of pneumothorax in our study had paraseptal emphysema provides clinical support for this association. It is important to note that only tobacco smoking history was systematically documented in this retrospective cohort. Epidemiological evidence increasingly supports the role of non-tobacco exposures—including biomass combustion, occupational dust, and air pollution—in the development of early-stage emphysema. In certain regions of Turkey, biomass smoke exposure remains prevalent and may have contributed to the structural lung changes observed, particularly among the 30.2% of never-smokers. Future prospective studies should systematically document non-tobacco exposures and incorporate them into multivariable analyses.

This study has several limitations that should be acknowledged. The retrospective, single-center design does not allow for the evaluation of causal relationships or longitudinal clinical course. All participants were recruited from a specialized tertiary referral clinic, introducing selection bias; the symptomatic burden and emphysema prevalence here likely overestimate those in the general young adult population. The predominance of male participants (84.1%) limits applicability to female individuals. The absence of a non-emphysema comparator group and a healthy control cohort precludes causal inference and the establishment of normative CT thresholds. Alpha-1 antitrypsin (A1AT) deficiency testing was not performed in this retrospective cohort. While A1AT deficiency is relevant to the etiology of emphysema, the primary aim of our study was to characterize the relationship between CT-detected emphysema extent and clinical outcomes within defined spirometric phenotypes, independent of etiology. Nevertheless, given the 30.2% never-smoker proportion and the presence of panacinar emphysema in 3% of patients, A1AT testing would have been informative for etiological characterization and should be incorporated into future prospective studies. Only tobacco smoking history was documented; non-tobacco exposures were not systematically recorded. Symptom assessment was limited to the mMRC dyspnea scale; multi-dimensional tools such as St. George’s Respiratory Questionnaire were not available due to the retrospective design. Finally, advanced pulmonary function measurements—including total lung capacity, residual volume, and diffusing capacity of the lungs for carbon monoxide (DLCO)—were not available, limiting a comprehensive functional assessment.

## 5. Conclusions

This study demonstrates that CT-detected emphysema in symptomatic young adults without airflow limitation is associated with statistically significant but weak alterations in pulmonary function and dyspnea burden. The PRISm phenotype was associated with significantly greater dyspnea severity compared with pre-COPD despite similar emphysema extent between the groups. The substantial proportion of never-smokers with CT-detected emphysema highlights the need for awareness of non-tobacco risk factors. Multicenter prospective studies incorporating healthy controls, and multivariable analyses are needed to confirm these findings and better characterize early emphysema pathophysiology in young adults without airflow obstruction.

## Figures and Tables

**Table 1:** Clinical and Demographic Characteristics of the Patients Included in the Study.

Variable	All Patients
**n**	232
**Age, years**	43 (38–48)
**Male sex**	195 (84.1%)
**Smoking history**	
Never smoker	70 (30.2%)
Former smoker	30 (12.9%)
≤10 pack-years	88 (37.9%)
10–20 pack-years	21 (9.1%)
≥20 pack-years	23 (9.9%)
**Patients with pre-COPD**	159 (68.5%)
**Patients with PRISm**	73 (31.5%)
**Comorbidity**	62 (26.7%)
Cardiovascular disease	13 (5.6%)
Hypertension	11 (4.7%)
Diabetes mellitus	10 (4.3%)
Obstructive sleep apnea syndrome (OSAS)	4 (1.7%)
History of pneumothorax	28 (12.1%)

Data are presented as median (interquartile range, 25th–75th percentile) or number (%). PRISm: Preserved Ratio Impaired Spirometry; COPD: Chronic Obstructive Pulmonary Disease; OSAS: Obstructive Sleep Apnea Syndrome.

**Table 2:** Emphysema Characteristics and PFT Results of the Patients Included in the Study.

Variable	All Patients
**n**	232
**Type of emphysema**	
Centrilobular emphysema	94 (40.5%)
Paraseptal emphysema	103 (44.4%)
Panacinar emphysema	7 (3.0%)
Centrilobular and paraseptal	26 (11.2%)
Bullous lung disease	2 (0.9%)
**Extent of emphysema (%)**	
Total lung emphysema area	18 (13–22)
Right lung emphysema area	17 (14–23)
Left lung emphysema area	18 (13–23)
**PFT parameters**	
FEV_1_ (L)	3.19 (2.98–3.83)
FEV_1_ (% predicted)	86 (77–96)
FVC (L)	3.93 (3.59–4.43)
FVC (% predicted)	84 (78–95)
FEV_1_/FVC	82 (77–87)
MEF 25–75 (% predicted)	81 (68–100)

Data are presented as median (interquartile range, 25th–75th percentile) or number (%). PFT: Pulmonary function test; FEV_1_: Forced Expiratory Volume in one second; FVC: Forced Vital Capacity; MEF 25–75: Maximal Expiratory Flow at 25–75% of forced vital capacity.

**Table 3:** Subgroup Comparisons According to the Degree of Emphysema.

Variable	Emphysema ≤ 18%	Emphysema > 18%	*p* Value
**n**	131 (56.5%)	101 (43.5%)	
**Age, years**	44 (38–49)	43 (37–48)	0.423
**Smoking history**			0.230
Never smoker	31 (23.7%)	39 (38.6%)	
Former smoker	16 (12.2%)	14 (13.9%)	
≤10 pack-years	55 (42.0%)	33 (32.7%)	
10–20 pack-years	12 (9.2%)	9 (8.9%)	
≥20 pack-years	17 (13.0%)	6 (5.9%)	
**Male sex**	107 (81.7%)	88 (87.1%)	0.262
**Body mass index** (kg/m^2^)			0.001
Underweight (<18.5)	28 (21.4%)	38 (37.6%)	
Normal weight (18.5–24.9)	86 (65.6%)	59 (58.4%)	
Overweight (25.0–29.9)	14 (10.7%)	2 (2.0%)	
Obese (≥30.0)	3 (2.3%)	2 (2.0%)	
**mMRC score**			0.007
0	57 (43.5%)	32 (31.7%)	
1	67 (51.1%)	50 (49.5%)	
2	7 (5.3%)	19 (18.8%)	
**Presence of comorbidity**	40 (30.5%)	22 (21.8%)	0.136
**FEV_1_ (L)**	3.39 (2.97–4.01)	3.13 (3.02–3.67)	0.023
**FEV_1_ (% predicted)**	86 (77–97)	84 (77–96)	0.950
**FVC (L)**	3.93 (3.50–4.44)	3.93 (3.59–4.43)	0.818
**FVC (% predicted)**	85 (77–95)	84 (78–94)	0.703
**FEV_1_/FVC**	82 (77–88)	82 (78–87)	0.623

Data are presented as median (interquartile range, 25th–75th percentile) or number (%). Continuous variables were compared using the Mann–Whitney U test; categorical variables were compared using the chi-square test. mMRC: Modified Medical Research Council dyspnea scale; FEV_1_: Forced Expiratory Volume in one second; FVC: Forced Vital Capacity.

**Table 4:** Comparison of Clinical Characteristics Between the PRISm and Pre-COPD Groups.

Variable	PRISm Patients	Pre-COPD Patients	*p* Value
n	73 (31.5%)	159 (68.5%)	
Age, years	44 (38–48)	43 (37–48)	0.542
Smoking history			0.349
Never smoker	27 (37.0%)	43 (27.0%)	
Former smoker	9 (12.3%)	21 (13.2%)	
≤10 pack-years	23 (31.5%)	65 (40.9%)	
10–20 pack-years	3 (4.1%)	18 (11.3%)	
≥20 pack-years	11 (15.1%)	12 (7.5%)	
Male sex	59 (80.8%)	136 (85.5%)	0.364
Body mass index			0.869
Underweight	22 (30.1%)	44 (27.7%)	
Normal weight	42 (57.5%)	103 (64.8%)	
Overweight	7 (9.6%)	9 (5.7%)	
Obese	2 (2.7%)	3 (1.9%)	
mMRC score			<0.001
0	10 (13.7%)	79 (49.7%)	
1	46 (63.0%)	71 (44.7%)	
2	17 (23.3%)	9 (5.7%)	
Presence of comorbidity	20 (27.4%)	42 (26.4%)	0.876
Extent of emphysema			0.429
≤18%	44 (60.3%)	87 (54.7%)	
>18%	29 (39.7%)	72 (45.3%)	

Data are presented as median (interquartile range, 25th–75th percentile) or number (%). Continuous variables were compared using the Mann–Whitney U test; categorical variables were compared using the chi-square test. PRISm: Preserved Ratio Impaired Spirometry; COPD: Chronic Obstructive Pulmonary Disease; mMRC: Modified Medical Research Council dyspnea scale.

**Table 5:** Comparison of Clinical, Radiological, and Pulmonary Function Characteristics Between Ever-Smokers and Never-Smokers.

Variable	Ever-Smokers	Never-Smokers	*p* Value
**n**	162 (69.8%)	70 (30.2%)	
**Age, years**	44 (39–48)	43 (36–48)	0.306
**Male sex**	140 (86.4%)	55 (78.6%)	0.135
**Body mass index**			0.734 *
Underweight	46 (28.4%)	20 (28.6%)	
Normal weight	100 (61.7%)	45 (64.3%)	
Overweight	13 (8.0%)	3 (4.3%)	
Obese	3 (1.9%)	2 (2.9%)	
**mMRC score**			**0.026**
0	55(34%)	34 (48.6%)	
1	86 (53.1%)	31 (44.3%)	
2	21 (13%)	5 (7.1%)	
**Presence of comorbidity**	44 (27.7%)	18 (25.7%)	0.820
**Type of emphysema**			0.051 *
Centrilobular emphysema	64 (39.5%)	30 (42.8%)	
Paraseptal emphysema	77 (47.5%)	26 (37.1%)	
Panacinar emphysema	4 (2.5%)	3 (4.3%)	
Centrilobular and paraseptal	22 (13.6%)	4 (5.7%)	
Bullous lung disease	2 (1.2%)	0	
**Extent of emphysema**			**0.014**
≤18%	100 (61.7%)	31 (44.3%)	
>18%	62 (38.3%)	39 (55.7%)	
**PFT Parameters**			
FEV_1_ (L)	3.14 (2.95–3.70)	3.51 (3.04–4.13)	**0.002**
FEV_1_ (% predicted)	86 (79–97)	85 (77–96)	0.414
FVC (L)	3.93 (3.59–4.45)	3.93 (3.63–4.34)	0.761
FVC (% predicted)	85 (78–96)	83 (77–94)	0.352
FEV_1_/FVC	83 (77–87)	81 (77–85)	0.389
**History of pneumothorax**	21 (13%)	7 (10%)	0.066

Data are presented as median (interquartile range, 25th–75th percentile) or number (%). Continuous variables were compared using the Mann–Whitney U test; categorical variables were compared using the chi-square test. * Fisher-Freeman-Halton exact test was applied due to small expected cell counts. mMRC: Modified Medical Research Council dyspnea scale; FEV_1_: Forced Expiratory Volume in one second; FVC: Forced Vital Capacity. Bold values indicate statistically significant results (*p* < 0.05).

## Data Availability

The original contributions presented in this study are included in the article. Further inquiries can be directed to the corresponding author.
